# Importance of Reference Muscle Selection in Quantitative Signal Intensity Analysis of T2-Weighted Images of Myocardial Edema Using a T2 Ratio Method

**DOI:** 10.1155/2015/232649

**Published:** 2015-06-21

**Authors:** Iacopo Carbone, Helene Childs, Ahmed Aljizeeri, Naeem Merchant, Matthias G. Friedrich

**Affiliations:** ^1^CMR Centre, Montreal Heart Institute, University of Montreal, Montreal, QC, Canada H1T 1C8; ^2^Department of Radiological, Onchological and Pathological Sciences, Sapienza, University of Rome, 00161 Rome, Italy; ^3^Stephenson CMR Centre, Libin Cardiovascular Institute of Alberta, University of Calgary, Calgary, AB, Canada T2N 2T9; ^4^Western College of Veterinary Medicine, University of Saskatchewan, Saskatoon, SK, Canada S7N 5B4; ^5^King Abdulaziz Cardiac Center, Riyadh 11426, Saudi Arabia; ^6^Department of Diagnostic Imaging, Libin Cardiovascular Institute of Alberta, University of Calgary, Calgary, AB, Canada T2N 2T9

## Abstract

*Objectives.* The purpose of our study was to identify the suitability of various skeletal muscles as reference regions for calculating the T2 SI ratio for a semiautomated quantification of the extent of myocardial edema with T2-weighted images.* Methods*. Thirty-four patients with acute myocardial infarction (MI) were enrolled. The extent of myocardial edema was determined by T2 SI ratio map, using 4 different muscles as reference: major and minor pectoralis, serratus anterior, teres minor-infraspinatus, and subscapularis. The size of myocardial edema as visually quantified was used as the standard of truth. The control group consisted of 15 patients with chronic MI. Intra- and interobserver variability were assessed.* Results*. Due to poor image quality four patients were excluded from the analysis. In acute MI patients, serratus anterior muscle showed the strongest correlation with the visual analysis (*r* = 0.799; *P* < 0.001) and low inter- and intraobserver variability, while the other muscles resulted in a significant interobserver variability. In contrast, the use of other muscles as a reference led to overestimating edema size.* Conclusions*. In acute MI patients, serratus anterior resulted to be the most reliable and reproducible muscle for measuring the extent of myocardial edema.

## 1. Introduction

Myocardial edema has evolved as a novel noninvasive tool for assessing the acuity of heart diseases in experimental [1–3] and clinical [4, 5] studies. Acute myocardial injury leads to increased tissue free water content, partly because of protein disruption as well as net inflow through leaky capillaries. Because free water has a much (about 40-fold) longer T2 relaxation time, the affected tissue has a higher signal intensity in T2-weighted (T2-w) images. This has been reported in experimental models [1, 2] and in patients with acute coronary syndrome [3], acute myocardial infarction (MI) [4], acute myocarditis [5, 6], and stress-induced Takotsubo cardiomyopathy [7]. Depending on the underlying pathology, myocardial edema can be a regional or diffuse process.

The most frequently used CMR sequence for assessing myocardial edema is the segmented Fast Spin Echo technique with a triple inversion recovery preparation, which suppresses the signal from blood flow and fat [8]. Beyond its T2 sensitivity, this sequence has been shown to be particularly useful for visualizing tissue edema [9]. In such images, myocardial edema can be evaluated qualitatively by visual estimation, using signal intensity cut-off values based on specific thresholds above the mean signal of remote myocardium, or by normalizing the signal to skeletal muscle for quantifying global edema, as proposed for acute myocarditis [5, 10, 11]. The latter approach, optimally obtained using a body coil, provides a signal intensity ratio, also referred to as the T2 signal intensity ratio (T2 SI ratio) and may overcome an important limitation of T2-weighted imaging: artifacts leading to an artificially low signal intensity of the tissue. The T2 SI ratio does not use low signal intensity areas as reference regions and thus is not sensitive to such artifacts. The selection of the skeletal muscle, in contrast, is under the discretion of the reader and thus is often a source of observer bias. Furthermore, different skeletal muscles may be more or less suitable as a reference.

We aimed to identify the suitability of various skeletal muscles as reference regions for calculating the T2 SI ratio using the edematous area measured visually on T2-weighted images as a standard of truth, in patients with acute myocardial infarction.

## 2. Methods 

### 2.1. Subjects

We retrospectively selected 34 consecutive patients presenting with acute, first-time STEMI, who underwent CMR at 1.5 T within 5 days from onset of symptoms (mean time to reperfusion was 337 minutes). Patients were excluded if they were clinically unstable or had severe arrhythmias or known contraindications to CMR. A diagnosis of MI was based on infarct-typical ECG changes combined with a >2-fold elevation of creatine kinase and/or positive troponin T. Infarct localization was performed via coronary angiography which identified the territory of the culprit vessel.

The control group consisted of 15 patients who underwent a CMR follow-up 5 months after the acute myocardial infarction (chronic MI). The time between the first and the second CMR scan was 160 ± 25 days. None of the 15 patients had any clinical events between the two CMR studies.

Patients gave written informed consent and the study was approved by our internal review board.

### 2.2. CMR Protocol

CMR examinations were performed using a 1.5 T MRI system (Magnetom Avanto, Siemens Medical Systems, Erlangen, Germany). Localization was performed by using real-time, breath-hold, and steady state free precession images of true anatomic axes of the heart.

The CMR protocol included cine steady state free precession (SSFP) CMR images for left ventricular (LV) function in a short axis orientation from base to apex, for a total of 8 to 10 slices. Cine SSFP images were obtained using the following parameters: repetition time (TR) 3.2 ms, echo time (TE) 1.07 ms, flip angle 65°, slice thickness 10 mm, no interslice gap, matrix 125 × 192, field of view ranging from 340 to 400 mm, and voxel size 1.7 × 1.7 × 10 mm. T2-w images were obtained using a breath-hold, triple inversion recovery sequence (TR 2 RR intervals; echo time (TE) 61 ms, inversion time (TI) 170 ms, and flip angle 180°) in a short axis plane (slice thickness 10 mm; no interslice gap; field of view 340 to 400 mm; matrix 166 × 256) using a body coil in the same slice orientation as the cine SSFP images.

### 2.3. Image Analysis

**Figure 1:**
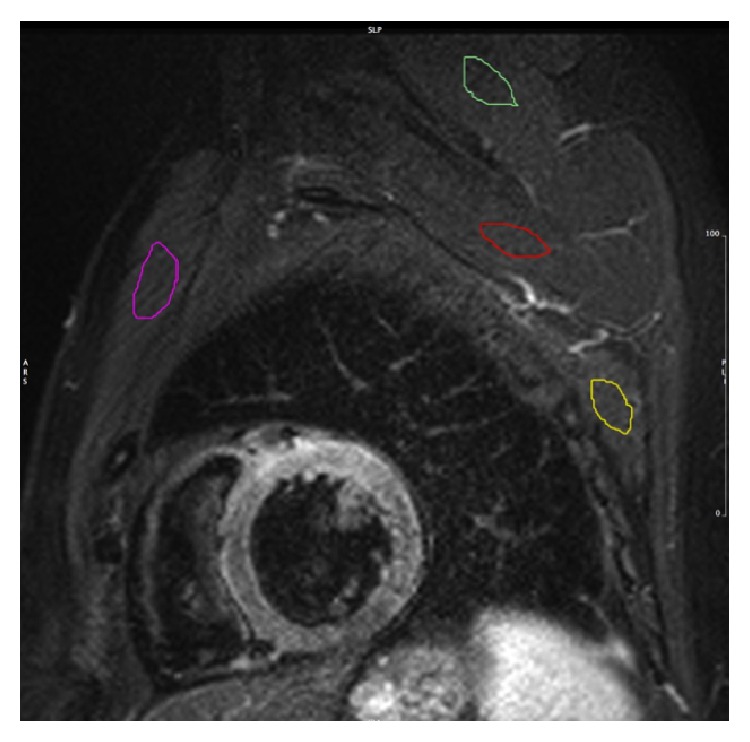
Short axis T2-w STIR image with 4 different ROI placed in 4 different skeletal muscles. The colored regions of interest mark the major and minor pectoralis muscles (purple), the serratus anterior muscle (yellow), the teres minor-infraspinatus muscle (green), and the subscapularis muscle (red).

The T2 SI in T2-w images was quantified with certified software (cmr^42^, Circle Cardiovascular Imaging Inc., Calgary, AB, Canada), using cine images of the same cardiac phase for verifying wall thickness and the correct identification of the skeletal muscle. The endocardial and epicardial borders of the LV myocardium were manually traced in each T2-w slice excluding trabeculae and papillary muscles. The T2 SI ratio was calculated by dividing the SI of the myocardium by the SI of skeletal muscle in the same slice [5]. In a color-coded map of the T2 SI ratio, at least 10 conjoint pixels with a positive T2 SI ratio were considered evidence of edema. A hypointense core within a high SI area was included in the edematous volume. Four different muscles were selected for T2 SI ratio analysis: one anterior, one posterior, and two superior (one proximal and one distal) to the LV myocardium (Figure 1). For the anterior muscle, a combination of major and minor pectoral muscles was used. The serratus anterior muscle (SA) served as the posterior muscle. For the superior muscle distal to the LV myocardium, a combination of infraspinatus (basal slices) and teres minor (apical slices) muscles was used. The subscapularis muscle was used for the superior muscle proximal to the LV myocardium. Using cine images as a reference each muscle group was identified on T2-w images and assessed for suitability based on the visible area and absence of fat.

Visual analysis of T2-w images was considered the reference standard for the quantification of the edema size. T2-w hyperintense areas were first defined by visual analysis during which the window settings could be freely adjusted to the personal preference of the observer and were manually contoured.

To test intraobserver variability the analysis of T2-w images was repeated in 20 randomly selected patients (10 patients with acute MI and 10 patients with chronic MI), after 4 weeks by the same reader (IC, 10 years of experience in CMR). To test interobserver agreement a second reader (AA, 1 year of experience in CMR) performed the T2 analysis separately in the same 20 patients (10 patients with acute MI and 10 patients with chronic MI).

### 2.4. Statistical Analysis

The difference between measurements was assessed with paired *t*-tests using PASW Advanced Statistics 19.0.0 (SPSS, Chicago). Differences were considered significant if the two-tailed *P* value was less than 0.05. Pearson correlation coefficient calculations were used to determine the linear relationship between sample observations with a significant correlation occurring when the *P* value was less than 0.05. Anova analysis was performed to compare edema mass average values. Bland Altman plots [12] were generated to assess inter- and intraobserver agreement by plotting the difference and mean values for each method. Intraclass correlation coefficient was computed to asses reproducibility both for intra- and interobserver measurements.

Chronic patient data were also analyzed using paired *t*-tests.

## 3. Results

### 3.1. Acute MI

We studied 34 patients, four patients were excluded due to poor quality T2-w images, leaving 30 patients (24 men; mean age 52.7 ± 11 years) with 168 T2-w images considered suitable for analysis. Detailed patients population characteristics are reported in Table 1. Edema location perfectly matched with angiographic findings and resulted to be anterior in 12 patients (40%), lateral in 3 (10%), and inferior in 15 patients (50%). A combination of major and minor pectoralis muscles was visualized in 164/168 images (97.6%); the serratus anterior was visualized in 150/168 slices (89.2%); the subscapularis was visualized in 131/168 slices (77.9%); a combination of teres minor and infraspinatus muscles was visualized in 135/168 slices (80.3%).

**Figure 2:**
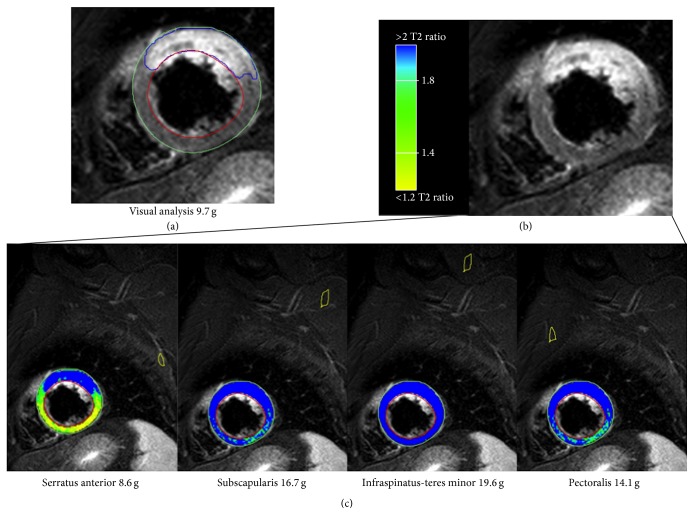
Results for quantitative assessment of the myocardium at risk in a patient with acute anterior myocardial infarction. (a) T2-w STIR image in a basal short axis view showing the quantification of the myocardial edema performed by the visual analysis, resulting in a mass of 9.7 g. (b) The same T2-w STIR without any contour. (c) Color-coded visualization of the automated sizing of the myocardial edema using the T2 SI ratio with four different muscle groups as reference regions. Despite a small overestimation, the serratus anterior resulted the most accurate for the size of the edematous myocardium (8.6 g).

**Figure 3:**
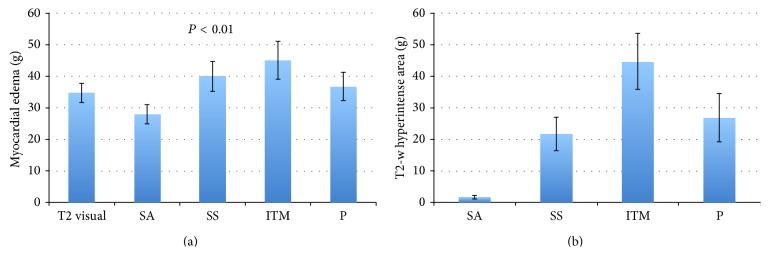
(a) Mean edema mass (±SE), obtained with the T2 SI ratio in different muscles in patients with acute MI. Increased T2-w SI area referenced to the serratus anterior muscle was closely correlated with myocardial edema measured visually. (b) Comparison between the mean T2-w hyperintense area (±SE), obtained with the T2 SI ratio, and other muscles in patients with chronic MI. False positive areas with increased T2-w SI area were very small, whereas with the use of other muscles they led to an apparent edematous area of more than 20 g. SA: serratus anterior; SS: subscapularis; ITM: infraspinatus-teres minor; P: major and minor pectoralis.

The mean size of edematous area in patients with acute MI using the visual analysis was 34.8 ± 3 g. Using the T2 SI ratio, the quantification of myocardial edema was 27.9 ± 3 g with the serratus anterior, 39.9 ± 4.7 g with the subscapularis muscle, 45.1 ± 5.9 g with a combination of infraspinatus and teres minor muscles, and 36.7 ± 4.5 g using a combination of major and minor pectoralis muscles (Figure 3(a)). Anova analysis resulted in a very low *P* value (<0.01) confirming that average values are different. Quantified visually, the edematous area was strongly correlated with the area determined by the T2 SI ratio, when the muscles serratus anterior, the subscapularis, and a combination of major and minor pectoralis muscles were used (*P* ≤ 0.001), while the serratus anterior showed the strongest correlation (*r* = 0.799) (Table 2, Figure 2). The area of increased T2-w SI measured by a combination of the teres minor and infraspinatus muscles was less correlated with the visual analysis (*P* = 0.008; *r* = 0.578). There was no significant difference between the extent of T2-w SI measured with the 4 different muscles and the visual analysis, although the serratus anterior showed a tendency to underestimate the edematous area (Table 2; Figure 3(a)).

For evaluations using the serratus anterior, the interobserver variability for the edematous area was low and there was no significant difference between readers (*P* = 0.142, Table 2). All other muscle groups resulted in a significant interobserver variability (*P* < 0.005, Table 2). Similarly, a low intraobserver variability was observed for high SI abnormalities as evaluated by the serratus anterior (*P* = 0.389, Table 2). While a combination of teres minor and infraspinatus also resulted in a very good agreement (*P* = 0.715, Table 2), the intraobserver variability was larger when a combination of major and minor pectoralis muscles or the subscapularis was used as a reference (*P* = 0.059 and 0.140, resp., Table 2).

**Figure 4:**
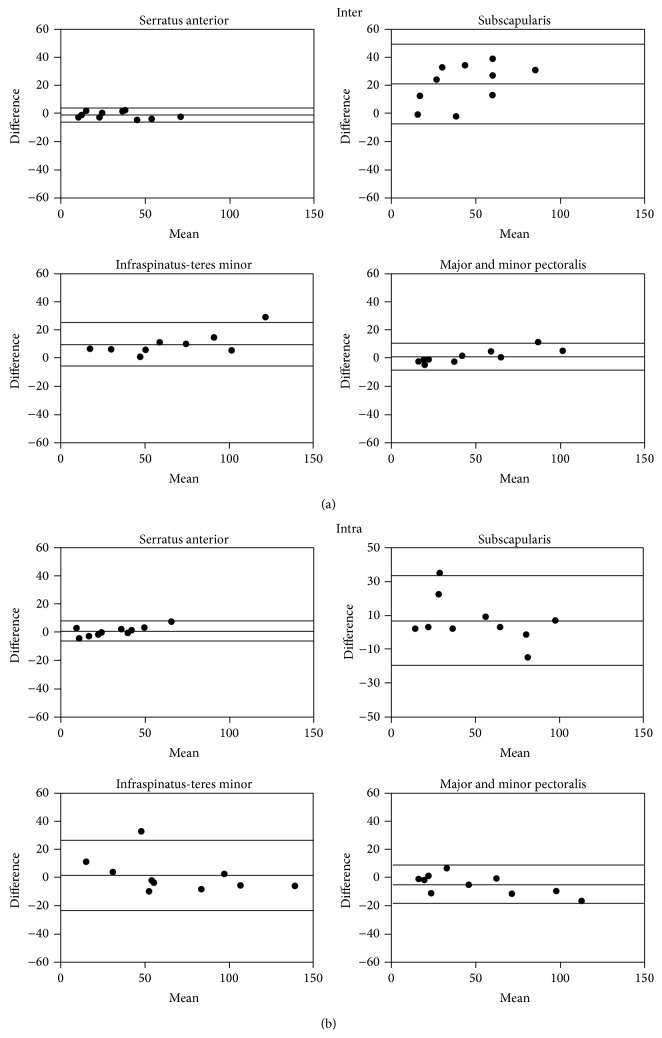
Bland Altman analysis of inter- (a) and intraobserver variability (b) of T2 SI ratio measurements with 95% confidence intervals using different muscle groups in acute MI in patients. Enhancement referenced to the serratus anterior muscle had the best inter- and intraobserver agreement.

A Bland Altman analysis indicated the serratus anterior muscle was far superior with respect to reproducibility of the edematous area in patients with acute MI. In both the intra- and interobserver analysis the serratus anterior measurements deviated least from one another, compared to other muscle groups used (Figure 4). The agreement mentioned earlier for the teres minor and infraspinatus group can also be visualized in these graphs (Figure 4). The subscapularis was the most unreliable muscle group (Figure 4).

### 3.2. Chronic MI

In the 15 patients with chronic MI, a total of 111 T2-w images were considered suitable for analysis.

A combination of major and minor pectoralis muscles was visualized in 99/111 slices (89.2%), muscle serratus anterior was visualized in 95/111 slices (85.6%), muscle subscapularis was visualized in 98/111 slices (88.3%), and a combination of teres minor and infraspinatus muscles was visualized in 95/111 slices (85.6%).

**Figure 5:**
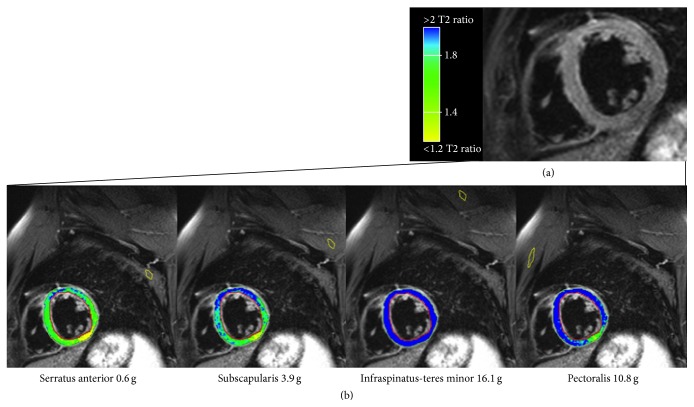
Basal short axis views in a patient 5 months after acute myocardial infarction (the same patient as shown during the acute stage in Figure 2). (a) T2-weighted image without evidence of significantly increased signal intensity. (b) Color-coded visualization of the automated sizing of the myocardium at risk using the T2 SI ratio with four different muscle groups as reference regions. All muscles except for the serratus anterior resulted in significant false positive results.

The mean size of the T2-w hyperintense area in patients with chronic MI was 1.5 ± 0.6 g using serratus anterior muscle, whereas for the subscapularis muscle, a combination of infraspinatus and teres minor muscles, and a combination of major and minor pectoralis muscles, it was 21.6 ± 5.2 g, 44.5 ± 8.9 g, and 26.7 ± 7.6 g, respectively (Figure 3(b)). An example of the analysis of chronic MI using all the different muscles is illustrated in Figure 5.

Using the muscle serratus anterior as a reference, there was no significant interobserver variability (*P* = 0.065, Table 3), while the subscapularis muscle was less reproducible between two readers (*P* = 0.039, Table 3). Using a combination of infraspinatus and teres minor muscles and a combination of major and minor pectoralis muscles also did not show any significant difference between observers (*P* = 0.580, *P* = 0.672, Table 3).

Also, no significant intraobserver variability was observed with the serratus anterior, infraspinatus-teres minor, or pectoralis muscle groups (*P* > 0.24, Table 3). There was, however, significant intraobserver variability when using the subscapularis muscle (*P* = 0.027, Table 3).

**Figure 6:**
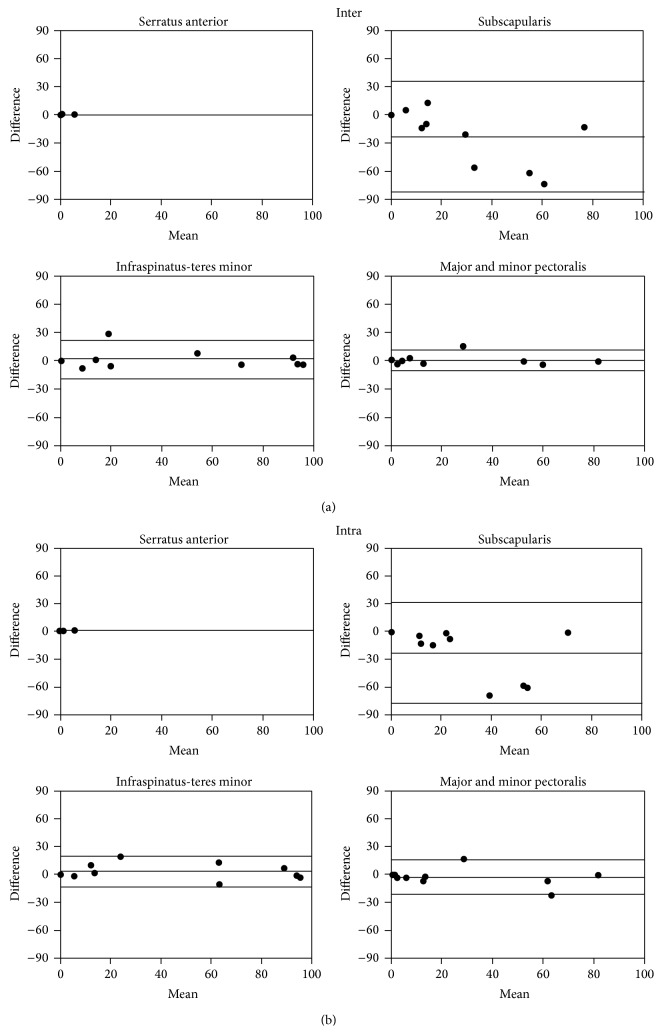
Bland Altman analysis of inter- (a) and intraobserver variability (b) assessment of the T2 SI ratio measurements of the edematous area with 95% confidence intervals using different muscle groups in patients with chronic MI. Enhancement referenced to the serratus anterior muscle had the best inter- and intraobserver agreement. 95% confidence intervals are labeled.

In patients with chronic MI, the Bland Altman graphs indicated that the serratus anterior was the best muscle due to its reliability and reproducibility with the same observer and between observers. In both the intra- and interobserver analysis the serratus anterior measurements deviated least from one another, compared to other muscle groups used (Figure 6).

## 4. Discussion

Our data indicate that the selection of skeletal muscles as reference regions affects results when quantifying the extent of the myocardial area at risk using the T2 SI ratio. The data indicate that sizing myocardial edema using the serratus anterior muscle as a reference resulted in the best agreement with its expected extent. While the area of the myocardium at risk strongly correlated with the visual quantification of the size of edematous area using all the 4 different muscles groups, the serratus anterior muscle had the strongest correlation and best interobserver and intraobserver agreement. The inferior correlation of other muscles may be explained by magnetic field inhomogeneities, affecting the signal intensity quantification in the context of a low signal-to-noise ratio.

The use of the serratus anterior muscle, however, led to an underestimation of the edematous area when compared with the visual assessment. This should be considered when an exact quantification of the salvaged area at risk is required (e.g., to assess the benefit of early [13] or late [1] revascularization).

In patients with chronic MI, where edema should not be present, the serratus anterior did not show relevant false positive results (apparent edema size 1.5 ± 0.6 g versus 21.6 ± 5.2 g to 44.5 ± 8.9 g when using other reference muscles). Furthermore in those patients, evaluation using the serratus anterior muscle as a reference also showed low intra- and interobserver variability. These results and the Bland Altman plots suggest that the serratus anterior is the best available reference muscle when normalizing the myocardial signal intensity for a quantitative analysis of myocardial edema.

Clear visibility of the muscle serratus anterior was present in 85–89% (85% in the chronic patients, 89% in the acute patients) and was mostly limited to the more basal planes. As per our results, however, a combination of the major and minor pectoralis can be used as an alternative to the serratus anterior muscle especially in the more apical planes.

While a standardized method for the quantification of myocardial edema appears essential, especially in patients with diffuse edema, most studies do not specify the skeletal muscle used for the T2 SI ratio [1, 5, 9, 14]. In a recent paper, Röttgen et al. [11] used the erector spinae when analyzing 131 patients with acute myocarditis; they reported a sensitivity and specificity of 58.3% and 57.1%, respectively, for the detection of edema using pathological specimen as a gold standard. A comparison with other reference muscles has not been performed, but the selection of the muscle may have had a significant impact on such analysis.

The exact cause of the better performance of the serratus anterior to other muscle groups could be related to B0 field inhomogeneity as well as the location of the muscle. Using the body coil, the isocenter of the magnet has the highest level of magnetic field homogeneity. With increasing distance from the center this homogeneity decreases, which may account for a signal loss in the skeletal muscle, leading to a false high T2 SI ratio. Similarly, there is also an increased B1 inhomogeneity related to the RF pulse in the periphery of the FOV. Furthermore, among the four muscle groups the serratus anterior was the only muscle located at the same level of the LV, along the *z*-axis, and thus is expected to be at the same level of homogeneity. Nonetheless, further studies are needed to confirm these findings and better understand the different variables involved in the assessment of edema using skeletal muscle referencing. Finally, with age, the subscapularis and teres minor-infraspinatus muscles tend to undergo atrophy and subsequent fatty replacement [14]. This would result in a decreased SI in the T2-STIR images and a subsequent increase of the T2 SI ratio when using these muscles as a reference.

Recently, T2 mapping has emerged as a novel approach to assess infarct-related edema [15]; yet the spatial resolution of T2 mapping is inferior to that of T2-weighted imaging and image registration issues may further limit its utility. Thus, more validation studies are required, before T2 mapping can be considered clinically useful.

### 4.1. Study Limitations

We used a visual analysis as a standard of truth in evaluating the area of increased T2-w signal intensity, which may be subject to observer bias. Visual analysis has been used as the standard reference for the present study, since myocardial edema cannot be histologically quantified in patients with AMI. An increased amount of free water results in a bright signal on T2-w images which can be visually seen.

While detection of focal processes (i.e., AMI) is more reliable with CMR, recognition of edema in diffuse diseases (i.e., acute myocarditis) remains a major challenge in which visual analysis is of limited support and validation of a reference muscle would provide better standardization and reproducibility of results [16].

The analysis was performed independently by an experienced observer, however, without comparing results during the analysis. Our sample is relatively small and represents patients with acute and chronic myocardial infarction only. Reflecting clinical practice, we analyzed images only in short axis views; thus, our results may be less applicable to long axis images.

## 5. Conclusion

This study shows that the serratus anterior muscle should be the first choice for quantifying the extent of myocardial edema by calculating the T2 signal intensity ratio with a skeletal muscle reference. This is due to the availability and reliability of the serratus anterior, relative to other muscles groups. However, more studies should be performed. Our findings also demonstrate the need for the standardization of T2 signal evaluation to avoid significant variability among different readers and centers.

## Figures and Tables

**Table 1:** Patients population characteristics.

Time to reperfusion (min)	335,73 ± 259,91
Troponin peak (UI/mL)	4,09 ± 5,10
TIMI after revascularization	3
Infarct-related artery	
LAD	12 (40%)
LCX	3 (10%)
RCA	15 (50%)

Risk factors	
Arterial hypertension	9 (30%)
Hypercholesterolemia	10 (33,3%)
Diabetes mellitus	3 (10%)
Familiar history of CAD	10 (33,3%)
Cigarette smoking	
Yes	12 (40%)
No	6 (20%)
Ex-smoker	12 (40%)

**Table 2:** Correlation of edematous mass as measured by the T2 SI ratio in patients with acute MI in different muscle groups with infarct mass (5SD LGE); differences as per paired *t*-test with corresponding *P* value and Pearson's correlation coefficient with *P* value. Intraclass correlation coefficient for interrater reliability and intrarater agreement.

Acute reperfused MI	Difference T2-w versus visual analysis	Correlation with T2-w visual analysis	Interobserver difference	Interobserver correlation	Intraobserver difference	Intraobserver correlation
Muscle	Mean (*P*)	*r* (*P*)	Mean (*P*)	ICC	Mean (*P*)	ICC
Serratus anterior	−6.8 ± 4.3 (0.118)	0.799 (<0.001)	1.23 ± 2.4 (0.142)	0.993	1.02 ± 3.6 (0.389)	0.981
Subscapularis	5.2 ± 5.6 (0.358)	0.669 (<0.001)	21.0 ± 15, (0.001)	0.521	7.0 ± 14 (0.140)	0.879
Teres minor/infraspinatus	10.3 ± 6.7 (0.129)	0.579 (0.008)	9.6 ± 7.9 (0.004)	0.933	1.52 ± 13 (0.715)	0.943
Pectoralis	1.9 ± 5.4 (0.715)	0.675 (<0.001)	1.44 ± 5 (0.001)	0.988	4.83 ± 7 (0.059)	0.972

**Table 3:** Extent of false positive edematous mass as measured by the T2 SI ratio in patients with chronic MI in different muscle groups; differences as per paired *t*-test with corresponding *P* value and intraclass correlation coefficient for interrater reliability and intrarater agreement.

Chronic reperfused MI	Interobserver difference	IIC interobserver correlation	Intraobserver difference	ICC intraobserver correlation
Muscle	Mean (*P*)	ICC	Mean (*P*)	ICC
Serratus anterior	0.30 ± 0.50 (0.065)	0.954	0.12 ± 0.36 (0.313)	0.98
Subscapularis	23 ± 30.0 (0.039)	0.328	23.2 ± 28.0 (0.027)	0.259
Teres minor/infraspinatus	1.91 ± 11.0 (0.580)	0.966	3.43 ± 8.6 (0.239)	0.974
Pectoralis	0.78 ± 5.6 (0.672)	0.983	2.95 ± 9.0 (0.349)	0.953

## Key Points

1. Role of skeletal muscle reference for the quantification of myocardial edema.
2. A standardized approach for the quantification of myocardial edema is highly desirable.
3. The muscle serratus anterior provides reliable and reproducible results for myocardial edema quantification.
